# *BDNF* DNA methylation changes as a biomarker of psychiatric disorders: literature review and open access database analysis

**DOI:** 10.1186/s12993-016-0101-4

**Published:** 2016-06-06

**Authors:** Galina Y. Zheleznyakova, Hao Cao, Helgi B. Schiöth

**Affiliations:** Department of Neuroscience, Uppsala University, Husargatan 3, BMC, 75124 Uppsala, Sweden; Department of Clinical Neuroscience, Karolinska Institute, Karolinska University Hospital, CMM L8:04, 17176 Stockholm, Sweden

**Keywords:** *BDNF*, DNA methylation, Biomarkers, Psychiatric disorders

## Abstract

Brain-derived neurotrophic factor (BDNF) plays an important role in nervous system development and function and it is well established that BDNF is involved in the pathogenesis of a wide range of psychiatric disorders. Recently, numerous studies have associated the DNA methylation level of *BDNF* promoters with certain psychiatric phenotypes. In this review, we summarize data from current literature as well as from our own analysis with respect to the correlation of *BDNF* methylation changes with psychiatric disorders and address questions about whether DNA methylation related to the *BDNF* can be useful as biomarker for specific neuropsychiatric disorders.

## Background

Brain-derived neurotrophic factor (BDNF) is a member of the neurotrophin family which plays an important role in neural differentiation, survival of nerve cells, neurite outgrowth, and synaptic plasticity. BDNF has been shown to regulate the development, plasticity and survival of dopaminergic, cholinergic and serotonergic neurons. Also, it regulates glutamatergic neurotransmitter release and promotes the development of GABAergic neurons. BDNF is widely expressed throughout the mammalian brain, including the cerebral cortex, hippocampus, basal forebrain, striatum, hypothalamus, brainstem, limbic structures and cerebellum [1]. This makes BDNF a key factor in learning and memory, reward-related processes, cognitive function and circuit formation.

The human *BDNF* has a complex gene structure, consisting of 11 exons (I–V, Vh, VI-VIII, VIIIh, IX), 9 of which (exon I–VII, IX) contain functional promoters. Exons II, III, IV, V, Vh, VI, and VIIIh do not have a translation start site so translation of these exons starts from the ATG of exon IX. All *BDNF* mRNAs contain the sequence for the pro-BDNF protein, encoded by exon IX. The use of translation start sites in exons I, VII, and VIII can lead to the pre-proBDNF proteins with longer N-termini. Alternative splice sites are situated in exons II, V, VI and in exon IX. In addition exon IX contains two alternative polyadenylation sites [2]. The use of different splice sites leads to the formation of numerous *BDNF* transcripts variants that determine a tissue-specific *BDNF* expression regulation as well as the regulation in responses to environmental stimuli and signaling events [2, 3]. mRNAs transcribed from the non-protein-coding anti*BDNF* gene and forming duplexes with *BDNF* mRNAs may play an important role in the regulation of *BDNF* expression [2]. Additionally, *BDNF* expression is regulated at the posttranscriptional level by enzymatic cleavage of the pro-BDNF protein into a mature BDNF protein. Pro-BDNF and BDNF can interact with two distinct transmembrane receptors, the p75 neurotropin receptor (p75NTR) and the tropomyosin-related kinase receptor B (TrkB). While pro-BDNF binds preferentially to p75NTR, inducing neuronal apoptosis and long-term depression, mature BDNF binds to TrkB and promotes a downstream signal cascade, leading to neuronal differentiation and survival, neurite outgrowth as well as synaptic plasticity [3, 4].

The BDNF protein has been extensively studied as an important factor involved in the pathogenesis of a wide range of psychiatric disorders, such as major depression, schizophrenia and bipolar disorders. The use of several animal models have also implicated BDNF in anxiety-like behaviors and have shown a decreased *BDNF* expression in response to different types of stressors [5, 6]. A Val66Met polymorphism (rs6265) of *BDNF* has been associated with different psychiatric conditions. This polymorphism is related to intracellular traffic, synaptic location, secretion of BDNF as well as poorer working memory performance, reduced cerebellar and hippocampal volumes, and cognitive ability [7, 8]. The polymorphism has been demonstrated to modulate a range of clinical features in schizophrenia patients. Higher frequency of the Met allele has also been associated with depression, anxiety, anorexia, bulimia nervosa and suicidal behavior [9, 10].

Numerous studies have addressed the association between psychological disturbances and levels of BDNF protein in serum or plasma. There are several sources of BDNF in blood: BDNF might be secreted by platelets, immune and vascular endothelial cells [11, 12]. BDNF is also able to overcome the blood–brain barrier [13]. With the correlations reported between BDNF levels in the brain and blood, altered BDNF levels has been established as characteristic for several psychological disorders. Low serum BDNF protein levels have been found in patients with depression, schizophrenia, anxiety and borderline personality disorder [14–17]. While certain treatments with antidepressants increased levels of BDNF in serum [14], a pre-treated level of BDNF has been shown to possibly predict the response to antidepressant treatment and has been correlated with depression rating improvements during therapy [18]. However, such clinical parameters as age of onset, the severity of symptoms as well as clinical effectiveness of the treatment were not reflected by BDNF levels in blood [14]. Additionally, proBDNF and the mature BDNF form cannot be discriminated by measurement in blood although they affect neuronal cells differently [16]. Since alterations of BDNF levels in blood associate with several psychological disorders, it cannot be considered as a specific biomarker for certain disease.

Epigenetic alterations as DNA methylation, histone modifications and non-coding RNAs are considered to be strongly associated with pathogenesis of psychiatric disorders (reviewed in [19, 20]) with a large number of studies having examined the association between the *BDNF* methylation level and certain psychological diagnoses. Epigenetic mechanisms have been shown to be very important for *BDNF* expression regulation [3, 4, 21, 22]. DNA methylation-related chromatin remodeling of *BDNF* regulatory regions may play critical roles in regulating gene transcription in response to neuronal activity [23, 24]. Epigenetic modifications, on the other hand, may potentially provide robust and stable biomarkers of disease activity. In this review, we have summarized the information about individual CpG sites and CpG regions previously tested in *BDNF* promoters and shown to be connected to psychiatric disorders. Using open access databases, we have analyzed the DNA methylation level of *BDNF* promoters in brain samples of individuals with psychiatric disorders. This review addresses the question of whether *BDNF* methylation changes might be regarded as suitable biomarkers of a specific disorder.

### A role of DNA methylation in gene expression

Epigenetic changes are gene expression and phenotype alterations heritable through cell division that are not caused by modifications in DNA sequences [25]. The main epigenetic mechanisms include DNA methylation, histone modifications and non-coding RNAs. DNA methylation is the first described and the most studied epigenetic modification [26]. Mammalian DNA methylation occurs predominantly in the nucleotide sequence 5′CpG3′. There are about 28 million CpG sites in the haploid human genome. CpG islands are defined as those sequences that have a length greater than 200 bp, a CpG content of at least 50 % and a CpG frequency greater than 0.6 of observed number to the expected number of CpGs [27]. The promoter regions of 60–70 % of all human genes contain CpG islands [26]. Traditionally high density CpG islands have been considered mostly hypomethylated [27, 28], however more recent studies have shown that dense CpG islands, depending on cell type, might be predominately hypermethylated [29]. A DNA methylation pattern is established and maintained by DNA methyltransferases (DNMT) namely de novo DNMT3A and DNMT3B and the maintenance DNMT1 [26]. DNA methylation affects gene expression, involving multiple mechanisms. Gene expression silencing is mediated by methylated DNA by attracting transcriptionally repressive methyl-CpG-binding proteins (MBPs). In turn, these proteins recruit histone deacytelases (HDACs) and chromatin-remodeling complexes, such as an NCoR-SMRT complex (nuclear receptor corepressor and silencing mediator for retinoid and thyroid hormone receptors), a NuRD (Nucleosome Remodeling Deacetylase) complex, which results in gene repression. The DNA methylation of a gene enhancer may also lead to gene repression. On the other hand, unmethylated CpG sites are bound by methyl-sensitive transcription factors, CXXC domain-containing activator complexes, thereby contributing to gene expression (reviewed in [30]). Unmethylated CpG sites might also serve as transcriptional factors “landing lights”, marking gene promoter regions which distinguishes them from the transcriptionally irrelevant intergenic chromatin [27]. Gene body DNA methylation may have a positive influence on gene transcription [31] and intragenic DNA methylation may affect alternative gene splicing [32]. Other interesting findings are the correlation between the density of gene-body DNA methylation and replication timing [30], and the influence of 5′UTR and 3′UTR DNA methylation on the elongation and termination of transcription [33].

Initially DNA methylation has been associated with prevention of particular gene expression. However, recent studies have introduced a more complicated impact of DNA methylation on gene expression regulation with several studies having identified a poor correlation between methylation levels of some genes and their expression level [27]. Differential DNA methylation of CpG islands associated with repressed genes has been found between somatic cells [27] which might imply stochastic DNA methylation of repressed genes. A few studies indicated that binding of transcription factors may precede DNA methylation changes in the enhancer, suggesting an inactive role of DNA methylation in enhancer activity [34]. Thus, DNA methylation is a complex epigenetic modification, which does not always determine gene expression activity. Several studies align with the idea that DNA methylation changes might be triggered by an antisense transcription, changes in histone modification and chromatin protein activity and thus might be a consequence rather than a reason for gene regulation [34–36].

Dynamic changes in DNA methylation are considered important mechanisms in the developmental regulation of gene expression. Embryonic stem cells are pluripotent and characterized by hypomethylation of CpG islands. During differentiation, genes essential for cell specification remain unmethylated while opposite genes, specific for other cell line development, are kept methylated. According to this notion, somatic cells demonstrate different DNA methylation patterns of a number of genes. A genome-wide analysis, comparing the DNA methylation profile across brain and blood, reveals highly tissue-specific differences in DNA methylation between different cortical regions, cerebellum and blood. It has also been shown that tissue-specific differentially methylated regions (TS-DMR) across the cerebellum and frontal cortex are associated with stable gene expression differences [37]. One of most interesting findings was an over-representation of intragenic CpG islands and an under-representation of promoter-associated CpG islands among TS-DMR. Low density CpG promoters were characterized by widespread tissue-specific DNA methylation across brain regions and blood, in comparison with CpG-rich promoters [37]. An intriguing recent study has also demonstrated a significant underrepresentation of promoter regions and an overrepresentation of CpG shores and shelves, gene bodies, as well as underrepresentation of CpG-rich promoters among fetal brain DMRs [38]. Another whole genome DNA methylation study has shown that similar pathways are affected in the brain and blood of Parkinson’s patients. Differently methylated regions between the blood and brain have been predominately presented in high-methylation fractions which are associated with gene bodies and intragenic regions [39].

5-hydroxymethylcytosine (5hmC), derived from oxidation of 5-methylcytosine (5mC) by the Ten-Eleven Translocation (TET) enzymes, is now considered a new epigenetic DNA modification with relevant roles in regulating DNA demethylation and transcription. 5hmC is generally associated with transcribed genes promoters and bodies, positively correlated with transcription levels and detected in the mammalian genome in all cell types, with the highest content present in the brain [40].

**Fig. 1:**
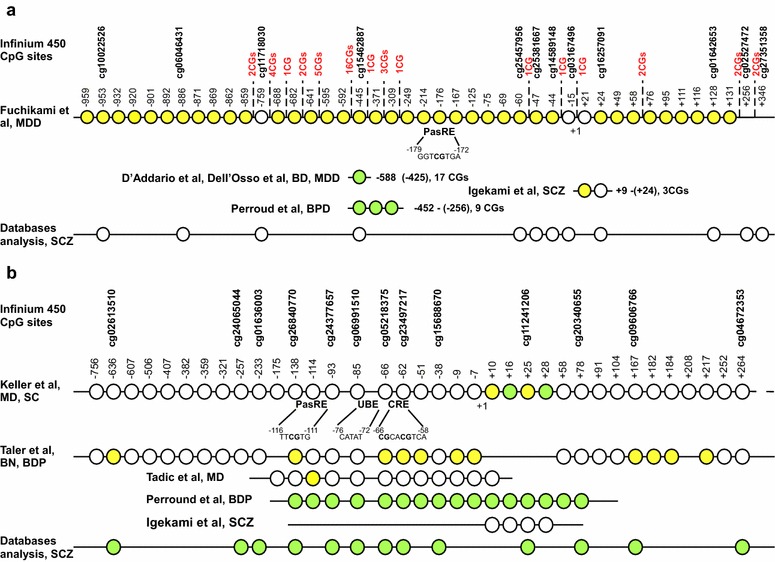
The positions of CpG sites analyzed in *BDNF* promoter I/exon I (**a**) and promoter IV/exon IV (**b**) in various studies. The CpG sites that are significantly differently methylated between cases and controls are marked *yellow*. The CpG sites, which are part of the significantly different methylated regions, are marked *green*. TSS is indicated by +1. Additional CpG sites inside of promoter I are highlighted *red*. The positions of the CpG sites on Infinium 450 K platform are shown. CRE, PasRE, UBE—cis-elements regulating neuronal activity-dependent transcription of *BDNF* promoters

The role of DNA methylation in the regulation of *Bdnf* expression was actively investigated by Martinowich and colleagues [23]. The authors found a three-four times higher level of the *Bdnf* exon IV transcript in Dmnt1 mutant mice in comparison with controls. It was also demonstrated that the activation of *Bdnf* transcription is regulated by neuronal activity. Enhanced transcription of the *Bdnf* exon IV promoter was observed in mouse embryonic cortical cells treated with 50 mM KCL, which is known to simulate neuronal activity by activation of voltage-sensitive calcium channels leading to calcium influx and membrane depolarization. More importantly, the authors identified that the region upstream of the *Bdnf* promoter IV transcriptional start site contains Ca^2+^-responsive elements, namely the calcium-responsive element 1 (CaRE1), an upstream stimulatory factor-binding site (E-box) and a cyclic adenosine monophosphate (cAMP) response element (CRE), which overlap with several CpG sites. A site-specific DNA methylation in combination with a luciferase activity test showed that DNA methylation of some of these CpG sites can significantly inhibit the *Bdnf* promoter IV activity induced previously by membrane depolarization. Further experiments showed that the methylation level of several CpG sites in the *Bdnf* exon IV was significantly lower in KCl-treated culture of mouse E14 cortical cells compared with control culture. This confirms that the methylation level of the *Bdnf* promoter IV can be changed upon depolarization. Subsequent chromatin immunoprecipitation analysis revealed that methyl-CpG-binding protein MeCP2 (transcription repressor) is more tightly associated with methylated DNA within the *Bdnf* exon IV than CRE-binding protein (transcription activator). Coimmunoprecipitation assay demonstrated that the histone deacytelase HDAC1 and corepressor mSin3A also associated with the *Bdnf* promoter. All three proteins dissociated from the promoter upon membrane depolarization suggesting that *Bdnf* expression activation requires the dissociation of the MeCP2-HDAC-mSin3A repression complex. All together, these findings indicated that *Bdnf* expression level is determined by the DNA methylation pattern and chromatin modifications which, in turn, can be regulated by membrane depolarization. In a latter study, the regulation of human *BDNF* transcription by membrane depolarization was confirmed [41]. The authors indicated that neuronal activity-regulated transcription of human *BDNF* promoter I depends primarily on the novel asymmetric E-box-like element, PasRE (basic helix-loop-helix (bHLH)-PAS transcription factor response element), which is bound by the bHLH-PAS transcription factors ARNT2 (aryl hydrocarbon receptor nuclear translocator 2) and NPAS4 (neuronal PAS domain protein 4). While neuronal activity-regulated transcription of the *BDNF* promoter IV is regulated predominately by CRE, PasRE elements and the upstream stimulatory factor binding element (UBE). The CRE and PasRE elements overlap with CpG sites (Fig. 1), however a separate study is necessary to investigate if DNA methylation of these CpG sites can influence transcription of the *BDNF* promoters following membrane depolarization.

Several studies in rats have demonstrated that stressful environment conditions, such as an early-life adverse experience or maltreatment, may induce long-lasting changes in the methylation level of the *Bdnf* promoter IV which is associated with the lower *Bdnf* expression level in prefrontal cortex. Moreover, the offspring derived from maltreated-females showed hypermethylation of the *Bdnf* in the prefrontal cortex and hippocampus, suggesting that DNA methylation modifications might be inherited across generations [42, 43].

### DNA methylation of *BDNF* in psychiatric disorders

DNA methylation alterations of *BDNF*, in connection to various psychiatric disorders, have been extensively studied in last several years (Table 1). Firstly, *BDNF* was studied by applying the enrichment microarray analysis; Mill and colleagues [44] tested the methylation level of several *BDNF* regions. They did not reveal any difference in the methylation level in patients with schizophrenia and bipolar disorders in comparison with controls. However, they identified an influence of rs6265 genotype, situated in exon IX on the methylation level of the surrounding region. Depending on the allele (C or T) of the rs6265, an additional CpG site can emerge in the region. CC (Val homozygotes) genotype carriers demonstrated a significant increase in methylation levels of four nearby CpG sites in comparison with CT and TT (Met homozygotes) genotype carriers. This is especially interesting, as several studies have shown the opposite effect of the Val/Val and Met/Met genotype on the schizophrenic and non-psychotic psychiatric disease phenotype [9]. In the recent study of Chagnon and colleagues, a significantly higher methylation level of one *BDNF* region (exon VI, 3 CpG sites) was observed in older women with anxiety and/or depression compared with controls [45]. This difference was more pronounced in CT genotype carriers of rs6265 in comparison with the CC genotype carriers. TT carriers were not found among both patients and controls. The higher DNA methylation in women with anxiety/depression compared with healthy controls was confirmed in a second small CT genotype carriers’ cohort (eight subjects). No difference was detected for CC and TT carriers. A comprehensive study assessing the whole *BDNF* methylation levels in a large cohort of CC, CT and TT carriers will be necessary to elucidate the impact of rs6265 on the methylation level of distal *BDNF* regions.

**Table 1:** Summary of human studies of the *BDNF* promoters’ DNA methylation in psychiatric disorders

Reference	Year	*BDNF* region	Chromosomal position (hg19)	Phenotype	Tissue	Samples	Method	Statistical power
Fuchikami et al.	2011	Promoter I Promoter IV	chr11:27743473–27744564 chr11:27722840–27723980	Major depression (MDD)	Peripheral blood	20 MDD, 18 CT	EpiTYPER	CpG8,9—75.7 % CpG76—54.3 % CpG80,81—79.1 % For rest CpGs—>80 %
D’Addario et al.	2012	Promoter I	chr11:27744031–27744193	Bipolar disorder I, II (BDI, BDII)	Peripheral blood	49 BD I, 45 BD II, 52 CT	Methylation-specific real-time PCR	BDII vs CT—62.5 % Antidep. vs antidep-free—93 %
D’Addario et al.	2013	Promoter I	chr11:27744031–27744193	Major depression	Peripheral blood	41 MDD, 44 CT	Methylation-specific real-time PCR	Methylation level —83.1 % Expression level—65.9 %
Ikegame et al.	2013	Promoter I Promoter IV	chr11:27743390–27743763 chr11:27722994–27723372	Schizophrenia (SCZ)	Peripheral blood	100 SCZ, 100 CT	Bisulfite pyrosequencing	CpG72—52.4 %
Perroud et al.	2013	Promoter I Promoter IV	chr11:27743862–27744057 chr11:27723057–27723293	Borderline personality disorder (BPD)	Peripheral blood	115 BPD, 52 CT	High resolution melt analysis	BPD vs CT—100 %
Dell’Osso et al.	2014	Promoter I	chr11:27744031–27744194	Bipolar disorder I, II, major depression	Peripheral blood	43MDD, 61 BD I, 50 BD II, 44 CT	Methylation-specific real-time PCR	MDD, BDII vs BDI and CT—100 %
Kleimann et al.	2015	Promoter I Promoter IV Promoter VI	chr11:27743416–27744782 (3 regions) chr11:27723103–27723511 chr11:27722216–27722863 (2 regions)	Treatment-resistant major depression (electroconvulsive therapy)	Peripheral blood	11MDD	Direct bisulfite sequencing	Remit. vs non-remit. 4 treatment sessions: 91–100 %
Chagnon et al.	2015	Promoter I Exon III Promoter VI/Exon VI	NS	Anxiety/major depression	Saliva	19 MDD, 24 CT	Bisulfite pyrosequencing	MDD vs CT—86.3 % CT gen. MDD vs CT gen. CT—88.3 %
Keller et al.	2010	Promoter IV	chr11:27723126–27723144 (4 CpG sites)	Major depression, suicide	Brain (Wernicke area)	44 SU, 33 CT	Bisulfite pyrosequencing, direct bisulfite sequencing, EpiTYPER	CpG1—98.1 % CpG3—96.3 % Average level—98.3 %
Kordi-Tamandani et al.	2012	Promoter IV	NS	Schizophrenia	Peripheral blood	80 SCZ, 71 CT	Methylation-specific PCR	Methylation level—99 % Expression level—94 %
Tadic et al.	2014	Promoter IV	chr11:27723103–27723380	Major depression (antidepressant treatment)	Peripheral blood	39 MDD	Direct bisulfite sequencing	NA
Thaler et al.	2014	Promoter IV	chr11:27722840–27723980	Bulimic nervosa (BN), Borderline personality disorder	Peripheral blood	64 BN(F), 32 CT	EpiTYPER	BN vs CT—in average for all CpG sites—99.8 %
Kang et al.	2013	Promoter VI	chr11:27721688–27721823	Major depressive, suicidal behavior (antidepressant treatment)	Peripheral blood	108 MDD	Bisulfite pyrosequencing	Previous suicidal attempt—76.7 % Suicidal ideation during treatment—83.6 and 96.6 %
Kang et al.	2015a	Promoter VI	chr11:27721688–27721823	Depression related to breast cancer	Peripheral blood	74 D, 235 CT	Bisulfite pyrosequencing	D vs CT within 1 week and 1 year in average—81.2 %
Kang et al.	2015b	Promoter VI	chr11:27721688–27721823	Late-life depression	Peripheral blood	101 D, 631 CT	Bisulfite pyrosequencing	D vs CT at baseline and after 2 years in average—97.7 %
Unternaehrer et al.	2015	Exon VI	chr11:27721543– 277221857	Low maternal care (LC) vs. high maternal care (HC)	Peripheral blood	45 LC, 40 HC	EpiTYPER	NA
Mill et al.	2008	Promoter IX	chr11:27679911–27680006	Schizophrenia, bipolar disorder	Brain (frontal cortex)	35 SZ, 35 BD, 35 CT	Enriched unmethylated DNA microarray, bisulfite pyrosequencing	Val homozygotes (78) vs Met carries (27)—100 %

Keller and colleagues were the first to investigate the *BDNF* DNA methylation changes in suicide victims in comparison with controls [46]. Using bisulfite pyrosequencing and direct bisulfite sequencing, EpiTYPER as a confirmation analyses, these authors analyzed the methylation levels of CpG sites at *BDNF* promoter/exon IV in the Wernicke area of the brain. A significant increase of the average methylation level of 4 CpG sites located downstream of TSS (+10, +16, +25, +28) (Fig. 1b) and methylation levels of two separate CpG sites (+10, +25) was found in suicide completers. Moreover, they also indicated an association between the average methylation level of these 4 CpG sites and the *BDNF* transcript IV levels.

Fuchikami and colleagues were the first to examine the possibility of DNA methylation changes as a biomarker of major depression. Applying the EpiTYPER technique, these authors determined the methylation level of two CpG islands associated with promoter I and promoter IV of *BDNF* in major depressive disorder (MDD) patients in comparison with healthy individuals. The methylation levels of 29 out of 35 CpG sites inside of promoter I (Fig. 1a) were significantly different between patients and controls, although different CpG sites demonstrated multidirectional methylation changes. 21 CpG sites showed increased methylation levels in controls, while the other 8 CpG sites showed increased methylation levels in depression patients [47].

In a study of major depressed patients with suicidal behavior, higher *BDNF* promoter VI methylation levels were found to be strongly associated with a previous history of suicidal attempts, suicidal ideation and less improvement during antidepressant treatment, independent of the antidepressant type [48]. As *BDNF* methylation status was not measured after the treatment in this study, it is not possible to conclude if treatment outcomes might be reflected by DNA methylation status. In two subsequent studies, authors found a significant association between the *BDNF* promoter VI methylation levels and late-life depression [49] as well as depression related to breast cancer [50]. It is worth mentioning, that both studies did not reveal any association between the methylation level of the analyzed region and rs6265 genotype.

Kleimann and colleagues, for the first time, examined an effect of electroconvulsive therapy (ECT) on DNA methylation of *BDNF* [51] in treatment-resistant major depressive patients. The difference in the methylation level of *BDNF* promoter I was presented between remitters/responders and non-remitters/non-responders over the whole series of ECT treatment. It is difficult to say if the found difference was conditioned by only ECT since there was no information about *BDNF* methylation level before ECT and most patients were previously treated by antidepressants and/or antipsychotics.

By means of bisulfite pyrosequencing, Igekame and colleagues found a significant increase (about 1 %) of the methylation level of one CpG site in the promoter I of *BDNF* in schizophrenia patients compared with controls [52] (Fig. 1a). Only in the male group of patients the additional CpG site was approximately 2 % higher methylated. It should be noted that the differences were not significant after multiple corrections and the sample size is not large enough to estimate a methylation level difference as low as 1–2 %. Authors did not reveal any difference in the methylation level of *BDNF* promoter IV.

On the contrary, Kordi-Tamandani and colleagues revealed that the methylated allele frequency of the *BDNF* promoter IV was lower in the schizophrenia patients, than in controls. According to the methylation data, the relative expression level of *BDNF* was significantly higher in schizophrenia patients than in controls [53].

In the study of D’Addario, a 7 % increase of DNA methylation at the region of *BDNF* promoter I (Fig. 1a) was observed in bipolar disorder (BD) II patients, but not BD I, compared with controls [54]. This was in correlation with the significantly decreased *BDNF* expression in BD II subjects. It should be taken in account that these data do not reflect the initial DNA methylation levels of BD patients, since part of the patients enrolled in the study were maintained on a diverse antidepressant treatment for at least 1 month. Interestingly, the authors showed, that BD patients on antidepressant treatment revealed a higher *BDNF* methylation level compared with antidepressants-free patients. However, the patients treated by valproate and lithium demonstrated a significantly decreased *BDNF* methylation level, in comparison with the control level.

In the subsequent study, these authors extended the *BDNF* promoter I methylation level analysis to the group of patients with major depression on stable pharmacologic treatment in comparison with healthy individuals [55]. In concordance with the previous study, a significantly increased methylation level of the analyzed region inside of *BDNF* promoter I was observed in MDD patients together with a significant reduction of *BDNF* expression. These authors also showed that MDD patients treated only by antidepressant drugs (i.e., selective serotonin and selective norepinephrine reuptake inhibitors) had a 10 % higher methylation level of the *BDNF* promoter in comparison with patients treated by combinatory therapy of antidepressants and mood stabilizers [55].

However, in a further study which was performed on a larger cohort of MDD, BD I and BD II patients, these authors were not able to show the effect of mood stabilizers on DNA methylation [56]. Patients treated by lithium and valproate tend to demonstrate *BDNF* methylation levels close to controls in comparison with patients treated by other antidepressant agents such as selective serotonin reuptake inhibitors, serotonin norepinephrine reuptake inhibitors and atypical antipsychotics. MDD subjects and BD II patients showed a significantly higher methylation level of the analyzed region in comparison with BD I subjects [56].

In the absence of an initial DNA methylation status for the patients, the results of these studies are difficult to interpret. It is not possible to discriminate if these described changes in the methylation level among different diagnostic groups are associated with disease status or whether they are connected with various pharmacological treatments. It was noted that BD I patients were mostly treated by mood stabilizers, while inside of the BD II and MDD groups, patients obtained a different therapy [56]. Thus, the *BDNF* promoter methylation analysis in drug-naive MDD, BD patients is necessary in order to make a reliable conclusion about whether this parameter might be used as biomarker.

Interesting results have been obtained in a study by Tadic and colleagues where the correlation between DNA methylation of *BDNF* promoter IV (Fig. 1b) in major depressive patients and antidepressant treatment response was assessed [57]. Of the 12 analyzed CpG sites, the methylation level of one CpG site was about 2 % lower in non-responders compared to responders. Following this, in vitro experiments showed a decreased luciferase expression of a vector containing an unmethylated fragment of *BDNF* promoter IV, in response to both SSRI and the SNRI treatment. These findings were connected with the ability of antidepressants to phosphorylate MeCP2, which leads to its dissociation from a promoter and consequently to the activation of expression. Moreover, these authors suggested that the DNA methylation status may play a significant role in the binding of MeCP2 to the promoter region and described that the mechanism of antidepressant action on *BDNF* can only be active in carriers of methylated allele at the specific CpG site. However, it is difficult to say that this conclusion was supported by experimental in vivo data, as both responders and non-responders demonstrated a strongly hypomethylated (4–6 %) status of the analyzed region.

DNA methylation levels of the *BDNF* promoter I and promoter IV (Fig. 1) were studied in patients with borderline personality disorder (BDP), whose disease phenotype is closely related to the depression and suicide phenotype. Using the high resolution melt (HRM) analysis, Perround and colleagues observed that regions in the *BDNF* promoter I and promoter IV had an almost 8 and 18 % higher methylation level respectively for BDP patients [58]. Moreover, a larger number of childhood maltreatment was significantly associated with a higher methylation status of *BDNF* promoters (mean percentage at both regions). Another very interesting result of this study is that during the intensive dialectical behavior therapy (I-DBT) non-responders showed increased *BDNF* methylation levels, while responders showed a decrease in *BDNF* methylation status, in some cases comparable to the methylation level of controls. This suggests that *BDNF* methylation changes might be relevant for treatment response prediction. The limitation of this study is that most of the subjects were on antidepressant treatment before I-DBT, that way the determined methylation level of BDP patients before treatment does not directly reflect a baseline methylation level of such patients.

An additional study of the influence of childhood maternal care on *BDNF* methylation level was performed by Unternaehrer and colleagues [59]. Authors showed greater whole blood DNA methylation in the low versus high maternal care group in a CpG island situated within the *BDNF* exon VI. More importantly, authors investigated differential blood cell distribution as a potential factor in connection with maternal care and DNA methylation of *BDNF*. It should be considered as an example of a standard experimental set up for such types of studies due to the blood cell specific DNA methylation pattern.

Thaler and colleagues explored DNA methylation changes of the *BDNF* promoter IV (Fig. 1b) in women with bulimic eating syndromes [60]. These women displayed increases in the methylation level at specific CpG sites, especially in cases when the bulimic syndromes were complicated by borderline personality disorder or history of severe childhood abuse. It is interesting that majority of found CpG sites were binding sites for various transcriptional factors. As no multiple corrections were applied, additional studies will be required to confirm the obtained differences.

## Open access study analysis

### Material

Four databases from the ArrayExpress Archive of Functional Genomics Data (http://www.ebi.ac.uk/arrayexpress) have been included in the analysis. Detailed information about each database can be found in Table 2.

**Table 2:** Open access analyzed databases

Database	Phenotype	Brain region	Groups	Age (years: mean ± SD)
E-GEOD-61107	Schizophrenia	Frontal cortex	23 SCZ: 7F, 16M	51.61 ± 21.55
			24 CT: 5F, 19M	71.29 ± 9.76
E-GEOD-61380	Schizophrenia	Frontal cortex	18 SCZ: 3F, 15M	45.5 ± 16.61
			15 CT: 2F, 13M	42.2 ± 14.85
E-GEOD-61431	Schizophrenia	Frontal cortex	20 SCZ: 9F, 11M	62.05 ± 15.87
			23 CT: 6F,17M	62.04 ± 18.74
E-GEOD-61431	Schizophrenia	Cerebellum	21 SCZ: 10F, 11M	61.76 ± 16.61
			23 CT: 6F, 17M	61.39 ± 19.25
E-GEOD-41826	Major depression	Frontal cortex: Split glial and neuronal cells	29 MDD: 15F, 14M	32 ± 15.92
			29 CT: 15F, 14M	32.1 ± 16.06

## Method

In the databases, the DNA methylation level was assessed at over 485,000 CpG sites using the Illumina Infinium Human Methylation450 Bead Chip. 25 probes corresponding to 12 CpG sites situated in the *BDNF* promoter I/exon I (Fig. 1a) and to 13 CpG sites situated in the *BDNF* promoter IV/exon IV were included in the analysis (Fig. 1b). The methylation level of each promoter was calculated by averaging the methylation levels of the corresponding CpG sites. Distribution normality for all variables was checked using Kolmogorov–Smirnov test. Because of non-Gaussian distribution, statistical comparisons of methylation levels between patients and controls were performed using the non-parametric Mann–Whitney test, although in the text and in the tables values are presented as mean ± SEM. A Bonferroni correction was used to adjust for multiple comparisons. Statistical analyses were performed using GraphPad Prism5 (GraphPad) and the statistical software R (http://www.r-project.org). A significance level of a = 0.05 or less was considered significant. The statistical power analysis for the described studies and the open access databases was performed with a web browser program “Post-hoc Power Calculator” (http://clincalc.com/Stats/Power.aspx#1).

**Table 3:** Methylation levels of *BDNF* promoters I and IV assessed in patients from open access databases

	Patients		Controls		p value	Statistical power
	Mean	SEM	Mean	SEM		
**Database E-GEOD-61107 (Schizophrenia, frontal cortex)**						
Promoter I	0.093	0.0026	0.099	0.002	0.09	
Promoter IV	0.113	0.0028	0.126	0.003	0.0015*	90.6 %
**Database E-GEOD-61380 (Schizophrenia, frontal cortex)**						
Promoter I	0.105	0.0009	0.108	0.002	0.0023*	30.4 %
Promoter IV	0.119	0.0012	0.12	0.0016	0.61	
**Database E-GEOD-61431 (Schizophrenia, frontal cortex)**						
Promoter I	0.123	0.0016	0.128	0.0017	0.13	
Promoter IV	0.157	0.0018	0.161	0.002	0.22	
**Database E-GEOD-61431 (Schizophrenia, cerebellum)**						
Promoter I	0.119	0.0027	0.117	0.0018	0.98	
Promoter IV	0.141	0.003	0.148	0.0029	0.0345	40 %
**Database E-GEOD-41826 (MDD, frontal cortex, neurons)**						
Promoter I	0.094	0.0007	0.093	0.0008	0.24	
Promoter IV	0.126	0.0009	0.125	0.001	0.39	
**Database E-GEOD-41826 (MDD, frontal cortex, glia)**						
Promoter I	0.106	0.001	0.107	0.001	0.30	
Promoter IV	0.125	0.001	0.126	0.001	0.32	

* Significant after multiple correction

## Results

According to results of previous studies both promoter I and IV of *BDNF* demonstrated strongly hypomethylated levels in all analyzed cohorts (Table 3). Frontal cortex methylation levels of both promoters I and IV were lower in schizophrenia subjects compared to controls (E-GEOD-61431, E-GEOD-61380, E-GEOD-61107 databases). Cerebellum methylation levels of promoter IV were lower in schizophrenia patients, at the same time cerebellum methylation levels of promoter I were similar in patients and controls. After applying multiple corrections, a significant difference in the methylation level of *BDNF* promoter I (p = 0.0023, Mann–Whitney test; E-GEOD-61380 database,) and of *BDNF* promoter IV (p = 0.0015, Mann–Whitney test; E-GEOD-61107 database) was found between schizophrenia patients and healthy individuals (Table 3). However, the power analysis indicated that the E-GEOD-61380 database is not powerful enough to determine a 0.3 % methylation level difference.

We did not observe any difference between MDD patients and controls in the methylation levels of *BDNF* promoters I and IV in the frontal cortex neuronal and glial cells (E-GEOD-41826). We separately analyzed several CpG sites which were determined as significantly differently methylated in previous studies (Fig. 1). The difference was found in the methylation level of cg14589148 (p = 0.0147, Mann–Whitney test; E-GEOD-61380 database), cg25457956 and cg10022526 (p = 0.0396 and p = 0.0227, Mann–Whitney test; E-GEOD-61431 database, cerebellum), however these data did not remain significant after multiple corrections.

## Discussion

The variety and complexity of psychiatric disorders is very high and biomarkers able to reliably differentiate characteristics of certain similar disorders, determine severity and treatment efficiency which could be very valuable. Taking into the consideration the inaccessibility of the target tissue—the brain—in psychiatric disorders, an ideal biomarker should be easily peripherally measured but at the same time it should reflect disease-associated alterations in the target tissue. DNA methylation is a stable and heritable modification and can be reliably measured from a small amount of material regardless of the storage conditions. These remarkable characteristics make DNA methylation a robust biomarker of disease activity. Numerous cancer studies have proven that DNA methylation changes allow manifestations of different pathological stages of disease and monitoring of treatment efficiency (reviewed in [61]).

At present, a growing body of data has associated *BDNF* promoters’ methylation level with development of various neuropsychiatric disorders. Most of the studies have analyzed different regions within promoter I and promoter IV. As it is demonstrated in Fig. 1a, the promoter I region is differently methylated in major depression and bipolar disorders patients in the studies done by D’Addario. These partly overlap with the region differently methylated in BPD patients of the Perroud’s study and with one CpG site differently methylated in major depression patients of the Fuchikami’s study. Similarly, within promoter IV, the CpG sites are differently methylated in suicide completers of Keller’s study, in patients with BDP and BN of the Taler’s study, and in major depressive patients in Tadic’s study, which partially overlap with the differently methylated region in BPD patients in Perroud’ study (Fig. 1b). These overlapping regions within *BDNF* promoter I and promoter IV may be of special interest as possible biomarker of psychiatric diseases, taking into consideration that several independent studies reported about the differences in the methylation level of these regions. Although the methylation levels’ alteration of these regions associated with multiple disorders do not allow the consideration of these methylation changes as reliable biomarkers for certain psychiatric disturbances.

Most of the studies have been performed using peripheral blood cells. The main question which appears is to what extent DNA methylation changes in blood can reflect the changes in the target tissue—the brain; a highly tissue-specified *BDNF* expression implies variation of *BDNF* promoter methylation level in various tissues. However, it has been reported that there might not be an exact correlation between gene DNA methylation and its expression level [27]. Moreover, all studies were concentrated on the methylation levels of *BDNF* promoters, which are CpG rich. It is well established that differently methylated regions across the brain and blood are primarily located in intragenic regions and are underrepresented in promoter and regulatory gene regions. CpG rich promoters were characterized by less tissue-specific DNA methylation across brain regions and blood [37, 39]. Recently, a strong correlation between the ventral prefrontal cortex and quadriceps for the methylation levels of *BDNF* promoter I has been found [62]. Similar epigenetic changes, particularly DNA methylation, in the brain and peripheral tissues in connection to psychiatric disorders might be initiated by environmental factors such as maternal stress or diet during early prenatal development or even have an inherited character. In the postnatal period, mothering behavior, early life traumas, environmental stressors factors and hormone dissonance may be responsible for the widespread epigenetic alterations associated with the psychiatric phenotype [19, 42, 43]. Keller and colleagues demonstrated that *BDNF* promoters’ DNA methylation changes in brain are associated with major depression and suicide. In one of the brain open-access databases, we revealed a significant but minor (1.3 %) difference in the average methylation level of *BDNF* promoter IV in schizophrenia patients.

Most of the changes found in the *BDNF* methylation levels between patients and healthy individuals are very subtle. The question about whether these differences may initiate changes in the *BDNF* expression levels has been partly addressed in previous studies. Thus, Keller and colleagues indicated much lower *BDNF* transcript IV levels in samples showing 20 to 30 % methylation of 4 CpG sites in *BDNF* promoter IV compared with samples showing 3 to 5 % methylation [46]. In the study of D’Addario and colleagues, a 9 % alteration in the *BDNF* promoter I methylation level between BDII patients and controls was matched with changes in the *BDNF* expression level in the blood cells [54]. It may suggest that the 9–15 % difference of the *BDNF* methylation level is functionally relevant and can induce changes in the *BDNF* transcripts level. In turn, Perroud and colleagues were not able to find any correlation between the *BDNF* promoters’ methylation and BDNF protein levels, although an 8 and 18 % methylation level difference was found respectively in *BDNF* promoter I and promoter IV between BDP patients and controls [58]. The discordance, in the association of the BDNF protein level with the DNA methylation level, might be conditioned by other regulators of *BDNF* expression: particularly histone modifications, miRNAs, antiBDNF transcripts, formation of dsRNA duplexes with *BDNF* transcripts, alternative splicing, and posttranscriptional cleavage. Recent studies have introduced the idea that DNA methylation may not even have an active influence on gene expression, but instead it might reflect gene expression regulation, mediated by histone modifications or chromatin structure [34]. Additionally, DNA hydroxymethylation, which cannot be distinguished from DNA methylation by bisulfite treatment, can impact *BDNF* expression in a manner opposite to DNA methylation. Therefore, a complex analysis of several factors determining *BDNF* expression is necessary to make the conclusion of whether the small changes in the *BDNF* methylation level may affect the *BDNF* expression changes both on transcript and protein levels.

Despite a relatively large number of studies, the use of the *BDNF* methylation level as a biomarker of psychiatric disorders still needs considerable further research to become reality. The main reason is the lack of confirmatory research for each particular disease. Another factor is the use of a variety of techniques for methylation level determination (Table 1), which hampers the analysis of different areas within the *BDNF* promoters without providing a correct comparison of the results of different studies for the same disorder. Although, methylation-specific real-time PCR and high resolution melt analysis are very sensitive methods with the ability to detect as little as 0.1–1 % of methylated DNA [63, 64], they cannot provide individual CpG sites’ resolution, allowing the determination of the average methylation level of a whole region. Primer design, PCR bias or the presence of too many CpG sites in the analyzed region might affect the obtained results. The bisulfite sequencing-based techniques widely applied in the studies differ also in their coverage and sensitivity. Direct bisulfite sequencing can accurately detect an intermediate difference (≥20 %) in methylation level while with pyrosequencing a minor difference of 5 % can be reliably detected while the resolution of cloning bisulfite sequencing is dependent on the number of sequenced clones [65, 66]. Furthermore, the inclusion of medicated and treated patients in the studies does not allow for specification of whether the observed DNA methylation changes are related to the specific disease phenotype or are a consequence of medical or psychiatric treatment. It is known that certain agents, applied for psychiatric therapy, cause epigenetic modifications [67]. Insufficient attention has been paid to *BDNF* methylation level investigations in the brain. So far only Keller’s study and our analysis indicate a partial correlation of the *BDNF* methylation level in the brain to the development of psychiatric disorders. However, we did not find any difference in the methylation level of the CpG site (cg11241206), differently methylated between controls and suicide completers in Keller’s study. Measured methylation levels of others CpG sites (Fig. 1), previously described as differently methylated in the studies, using blood cells did not show any correlation after multiple corrections. The restrictions of the method did not allow us to completely assess the methylation level of the regions analyzed in previous studies using brain samples as Infinium HumanMethylation450 BeadChip Kit covers far from all CpG sites in *BDNF* promoter I and IV (Fig. 1). A detailed analysis in the brain with a method, determining the methylation levels of all CpG sites, is required. Both Keller’s and our study covered only certain brain areas (frontal cortex, cerebellum, Wernicke area), while *BDNF* DNA methylation changes in such brain regions as hippocampus, nucleus accumbens, amygdala [3, 68, 69] may be essential for psychiatric disorders development.

## Conclusions

Summing up the results of this research provides a good reason to believe that the methylation levels of certain regions within *BDNF* promoters can be considered as biomarker of specific diseases. Several independent studies on different patients’ cohorts with a standardized method for the methylation level analysis are necessary to confirm this suggestion. Applying the most modern approaches for the analysis of methylation will allow highlighting of the role of both DNA methylation and DNA hydroxymethylation (primarily occurring in the neuronal cells) with regard to the development of mental illnesses. Considering the easy accessibility of blood samples, it is the DNA methylation level of blood cells that might be used in clinical practice for accurate disease diagnosis and treatment-efficacy monitoring. The question of DNA methylation discrepancy within various blood cell lines [70] and possible differences of cell composition between psychiatric patients and healthy individuals [71] should also be taken in account. Modern techniques of cell sorting and counting will help to smooth over such differences. Considering the fact that most changes in DNA methylation linked to mental illness are fairly small in terms of percentage, it is necessary to use a high-resolution method that can reliably detect small differences. We suggest that through further investigations, an unequivocal answer if the *BDNF* DNA methylation level is a suitable psychiatric disorder biomarker may be provided.

## Abbreviations

ARNT2: aryl hydrocarbon receptor nuclear translocator 2
BD: bipolar disorder
BDNF: brain-derived neurotrophic factor
BDP: borderline personality disorder
CaRE1: calcium-responsive element 1
CRE: cAMP response element
DNMT: DNA methyltransferase
ECT: electroconvulsive therapy
HDACs: histone deacytelases
HRM: high resolution melt
I-DBT: dialectical behavior therapy
MBPs: methyl-CpG-binding proteins
MDD: major depressive disorder
MSP: methylation-specific real-time PCR
NCoR: nuclear receptor corepressor
NPAS4: neuronal PAS domain protein 4
NuRD: nucleosome remodeling deacetylase
p75NTR: p75 neurotropin receptor
PasRE: basic helix-loop-helix transcription factor response element
SMRT: silencing mediator for retinoid and thyroid hormone receptors
SNRIs: serotonin norepinephrine reuptake inhibitors
SSRIs: selective serotonin reuptake inhibitors
TET: ten-eleven translocation enzymes
TrkB: tropomyosin-related kinase receptor B
TS-DMR: tissue-specific differentially methylated region
UBE: upstream stimulatory factor binding element
